# Bioassay Guided Isolation and Docking Studies of a Potential β-Lactamase Inhibitor from *Clutia myricoides*

**DOI:** 10.3390/molecules25112566

**Published:** 2020-05-31

**Authors:** Mahmoud A. Elfaky, Ali M. El-Halawany, Abdulrahman E. Koshak, Khalid Z. Alshali, Moustafa E. El-Araby, Maan T. Khayat, Hossam M. Abdallah

**Affiliations:** 1 Department of Natural Products and Alternative Medicine, Faculty of Pharmacy, King Abdulaziz University, Jeddah 21589, Saudi Arabia; melfaky@kau.edu.sa (M.A.E.); aekoshak@kau.edu.sa (A.E.K.); 2 Department of Pharmacognosy, Faculty of Pharmacy, Cairo University, Cairo 11562, Egypt; ali.elhalawany@pharma.cu.edu.eg; 3 Department of Medicine, Faculty of Medicine, King Abdulaziz University, Jeddah 21589, Saudi Arabia; kalshali@kau.edu.sa; 4 Department of Pharmaceutical Chemistry, Faculty of Pharmacy, King Abdulaziz University, Jeddah 21589, Saudi Arabia; madaoud@kau.edu.sa (M.E.E.-A.); mkhayat@kau.edu.sa (M.T.K.)

**Keywords:** β-lactamase, *Clutia myricoides*, *Klebsiella pneumoniae*, antimicrobial resistance, bio-guided fractionation, molecular docking

## Abstract

Infectious diseases are the second major cause of death worldwide, and the ability to resist multiple classes of antibiotics is the key factor in enabling pathogenic organisms to survive and spread in the nosocomial environment. Unfortunately, the available β-lactamase inhibitors are not efficient against β-lactamase B, C, and D which necessitates discovering either broad spectrum β-lactamase inhibitors or new β-lactam antibiotics resistant to bacterial enzymes. In this regard, products of natural origin have prompted the disclosure of new compounds and medicinal leads. Chloroform fraction of *Clutia myricoides* (Soa’bor) showed a pronounced activity against extended-spectrum β-lactamase (ESBL) strains. Bio-guided fractionation resulted in isolation of two new compounds; 2-methoxy chrysophanol (**2**) and Saudin-I (**5**) in addition to three known compounds that were identified as chrysophanol (**1**), stigmasterol (**3**) and β-sitosterol (**4**). Antibacterial and anti ESBL activities of the isolated compounds were performed. No antibacterial activities were detected for any of the tested compounds. Meanwhile, compound **2** showed promising anti ESBL activity. Compound **2** has shown an obvious activity against *K. pneumoniae* ATCC 700603 with a marked enlargement of inhibition zones (>5mm) in combination with third generation cephalosporin antibiotics. To further understand the mechanism of action of compound **2**, molecular docking was carried out against CTX-M-27 ESBL. The results showed binding site interactions strikingly different from its analogue, compound **1**, allowing compound **2** to be active against ESBL. These results proposed the concomitant use of these active compounds with antibiotics that would increase their efficiency. Nevertheless, the interaction between this active compound and antibiotics should be taken into consideration. Therefore, in order to evaluate the safety of this active compound, further in vitro and in vivo toxicity assays must be carried out.

## 1. Introduction

Contagious diseases are the second significant reason for death around the world. The capacity to resist various classes of antimicrobial agents is a key factor that enables the survival of pathogens in nosocomial settings. Management of *Acinetobacter baumannii* and *Pseudomonas aeruginosa*, which are connected to severe infections, is greatly troublesome as a result of their resistance to antimicrobials by various mechanisms [1]. *Klebsiella pneumoniae* is a serious microbe related with numerous hospitals acquired infections while the level of β-lactamase producing *Escherichia coli* began to wind up progressively harder to manage. Presence of *bla*CTX-M, *bla*SHV, and the *bla*TEM genes among the β-lactamase producing *K. pneumoniae* and *E. coli* are accounted for around the world [2]. The β-lactamases are the principal mechanism of resistance to β-lactam antimicrobials [3]. On the other hand, Gram-negative microorganisms are actually more unaffected by antibiotics than Gram-positive ones. This is attributable to transmembrane efflux [4].

Resistance of these bacteria to antibiotics containing β-lactam ring is mitigated by combination of these antibiotics with β-lactamase inhibitors. Many well-known examples are marketed such as, combination of clavulanic acid or sulbactam as β-lactamase inhibitors with antibiotics [5]. There is a critical need to a new and safer antimicrobial agent without cross-resistance as that available. Unfortunately, the available β-lactamase inhibitors are not efficient against β-lactamase B, C, and D which necessitates discovering either broad spectrum β-lactamase inhibitors or new resistant β-lactam antibiotics to bacterial enzymes.

Products of natural origins have prompted the disclosure of new compounds and medication leads [6]. Previous work has been achieved on the ability of some plants to inhibit β-lactamase enzyme and augment the effect of antibiotics [7]. Many researchers reported potentiation of phyto-compounds and antibiotic effect by plant extracts [8,9,10]. In a screening of a group of anti-bacterial Indian plants, *Punica granatum* and *Delonix regia* showed high activity against β-lactamase [10]. Moreover, previous work reported antibacterial activity of *Garcinia kola* against multidrug resistance extended-spectrum β-lactamase (ESBL) positive *Escherichia coli*. In addition, volatile oil constituents from some *Artemisia* species showed antimicrobial activity against ESBL-producing *E. coli* and augmented the action of antimicrobials [11]. Myricetin, a flavonol, inhibited ESBL-producing *K. pneumoniae* isolates at a high minimum inhibitory concentration (MIC) (MIC_90_ value 256 mg/mL), but exhibited significant synergic activity against ESBL-producing *K. pneumoniae* in separate combination with amoxicillin/clavulanate, ampicillin/sulbactam, and cefoxitin [12].

Genus *Clutia* (Euphorbiaceae) is native to the Arabian Peninsula [13]. The genus has not been investigated extensively but it is characterized by the presence of secolabdane-type diterpenes [14,15,16,17,18,19,20], coumarins [21], and anthraquinones [13]. In a previous work, nineteen plants belonging to eight families from Saudi flora were screened for their activity against ESBL strains of *K. pneumoniae* and other medically important pathogens [22]. Chloroform fraction of *Clutia myricoides* (Soa’bor) showed a pronounced activity against ESBL strains. Phytochemical screening of *C. myricoides* [23] revealed the presence of anthraquinone, cardiac glycosides, saponins, flavonoides, coumarins, condensed tannins, triterpenoids, steroids, and alkaloids. In contrast, essential oils and hydrolysable tannins were absent.

In this work, the bioactive compounds in the chloroform fraction of *C. myricoides* are isolated, identified, and tested for their activity against ESBL strains of *K. pneumoniae.*

## 2. Results and Discussion

### 2.1. Chemical Investigation of the Chloroform Fraction

**Figure 1:**
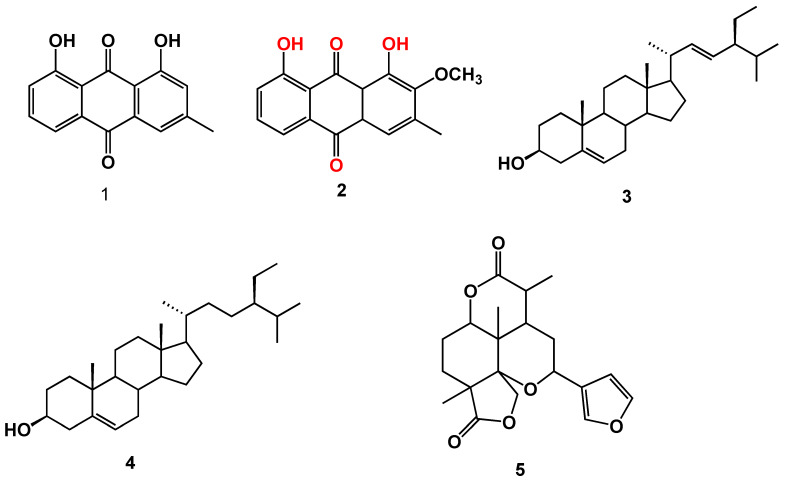
Structure of isolated compounds (**1**–**5**).

The air-dried aerial parts of *C. myricoides* were extracted with MeOH. The MeOH extract was suspended in the least amount of water and partitioned with CHCl_3_. The CHCl_3_ fraction was repeatedly chromatographed on SiO_2_ columns to furnish two new compounds (**2** and **5**) (Figure 1) and three known compounds that were identified as chrysophanol (**1**) [24], stigmasterol (**3**) [25], and β-sitosterol (**4**) [26]. All isolated compounds were identified based on their NMR spectral data (Figures S1–S11).

Compound **2** was obtained as yellow amorphous powder. Its molecular formula was determined as C_16_H_12_O_5_ on the basis of the HRESIMS pseudo-molecular ion peak at *m/z* 285.0769 [M + H]^+^ (calcd for 285.0763, C_16_H_13_O_5_).

^1^H-NMR spectrum (Table 1) extirpated one aromatic singlet at δ_H_ 7.69, two aromatic *meta* coupled protons as doublet of doublet (*J* = 1.7, 7.6 Hz) at δ_H_ 7.31 and 7.83, and one *ortho* coupled proton at δ_H_ 7.70 (*J* = 7.6 Hz). Moreover, the spectra displayed singlet because of the methoxy group at δ_H_ 3.99 and singlet for a methyl at δ_H_ 2.44. In addition, two hydroxyl aromatic protons were displayed as singlet at δ_H_ 12.37 and 11.90. ^13^C-NMR spectrum of **2** (Table 1) showed two carbonyl carbon signals at δ_C_ 192.4 and 181.4 in addition to signals because of 12 aromatic/olefinic carbons (of which three were oxygenated; δ 166.4, 162.6, and 159.7), and four protonated carbons (δ 120.3, 121.5, 124.9, and 137.4), a methoxy carbon (δ 52.8), and a methyl carbon (δ 20.1). These data suggested that **2** is a 9,10-anthraquinone bearing two hydroxyls, one methoxy, and a methyl substituent.

**Figure 2:**
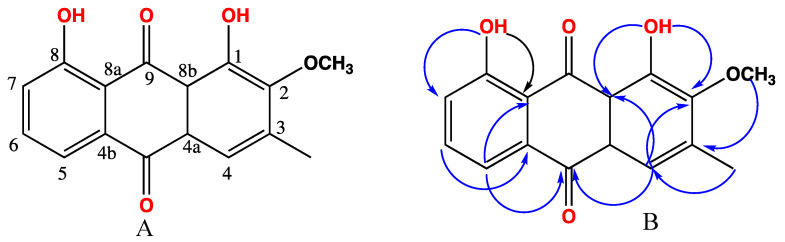
Structure of compound **2** (**A**) and some key HMBC correlations (**B**).

The substitution pattern was determined by the analysis of HMBC (Heteronuclear Multiple Bond Correlation) spectra (Figure 2). Placement of methoxyl group at C-2 was performed after the observed strong cross peaks between proton of methoxyl at δ_H_ 3.99 and carbons at δ_C_ 166.4, 159.1, and 146.4 for C-2, 1, and 3 respectively. Meanwhile, the methyl group was confirmed to be at C-3 after a strong correlation between δ_H_ 2.44 and carbons at δ_C_ 166.4, 146.4, and 121.5 for C-2, 3, and 4, respectively.

On the basis of the above evidences, the structure of **2** could be identified as 2-methoxy chrysophanol which was prepared synthetically [27] but have not been isolated from natural source previously. 

Compound **5** was obtained as white amorphous powder. Its molecular formula was determined as C_20_H_24_O_6_ on the basis of the HRESIMS pseudo-molecular ion peak at *m/z* 361.1676 [M+H]^+^ (calcd for 361.1670, C_20_H_25_O_6_).

^1^H-NMR spectra exhibited three downfield signals at δ_H_ 7.54 (Brs, H-2′), 7.40 (Brs, H-5′), and 6.45 (Brs, H-4′) (Table 1) that are correlated in HSQC with carbons δ_C_ 139.4, 144.8, and 107.0 respectively, indicating the presence of furan ring [18]. In addition, the spectra displayed a signal at δ_H_ 5.11 (d, *J* = 1.7, H-5), which is correlated with carbon at δ_C_ 92.0 that is characteristic for a proton at carbon adjacent to oxygen of tetrahydropyran [18]. Moreover, the presence of lactone rings, which is characteristic for compounds isolated previously from this plant, was confirmed through the presence of two protons displayed at 4.18 (d, *J* = 9.4, H-13) and 4.28 (d, *J* = 9.4, H-3b) for methylene group in γ-butyrolactone and presence of proton δ_H_ 4.37 (dd, *J* = 4.2, 11.9, H-9a) for δ-valerolactone. Moreover, the spectra exhibited three methyls, one doublet at δ_H_ 1.41 (d, *J* = 6.8), and two singlets at 1.43 and 1.20 for methlyls at δ_C_ 13.7, 23.0, and 15.3, respectively.

The aforementioned data confirm that compound **5** follows secolabdane diterpene which is characteristic for this plant [18]. The secolabdane diterpene skeleton was further confirmed through ^13^C-NMR that showed 20 carbon signals (Table 1) including signals corresponding to the furan ring (δ_C_ 107.0, 121.0, 139.4, and 144.8), two lactone carbonyls (δ_C_ 178.0 and 173.0; for C-1 and C-8 respectively), one highly oxygenated quaternary center (δ_C_ 82.0; C-3a), one oxygenated methine (δ_C_ 92.0 for C-5), and one oxygenated methylene (δ_C_ 70.4 for C-3).

**Figure 3:**
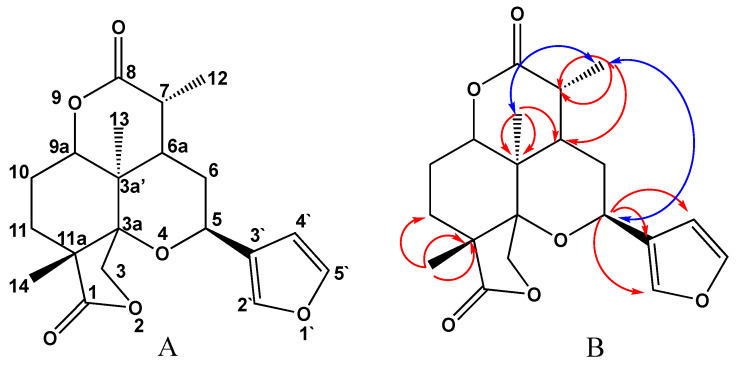
Structure of compound **5** (**A**) and some key HMBC and NOESY correlations (**B**).

The positioning of furan ring and methyls was confirmed from HMBC correlations (Figure 3). Furan ring was placed at C-5 after cross peaks between H-5 at δ_H_ 5.11 and carbons 2′, 3′, and 4′ at δ_C_ 139.4, 121.0, and 107.0 respectively. A doublet methyl at δ_H_ 1.41 was attached to C-7 (δ_C_ 35.3) after cross peaks in HMBC between this methyl and carbons at δ_C_ 35.3 (C-7), 173.0 (C-8), and 38.4 (C-6a). Also, a strong correlation in COSY between doublet at δ_H_ 1.41 and protons at δ_H_ 3.02 (H-7), δ_H_ 2.7 (H-6a) confirmed this positioning. Moreover, singlet methyl at δ_H_ 1.20 was positioned on C-3a (δ_C_ 35.2) after strong correlation in HMBC with carbons δ_C._ 35.2, 38.4, and 75.5, (C-3a’, 6a, and 9a respectively). Finally, singlet methyl at δ_H_ 1.43 was positioned on C-11a after strong correlation in HMBC with carbons δ_C_ 178.0, 44.0, and 26.5 (C-1, 11a, and 11, respectively). In addition, relative stereochemistry could be detected from NOESY correlations (Figure 3) and by referring to published data [19]. H-7 was reported to be at δ_H_ 2.9 and δ_C_ 47.4 if it is present in alpha position. In compound **5** this proton is displayed at δ_H_ 3.02 and δ_C_ 35.3 which confirm β configuration of H-7 and placed 12-methyl in alpha position. Methyl at 12 showed strong correlations in NOESY with H-5, and methyl at 13 that positioned these groups as alpha configuration, while furan ring attached to C-5 will be in β position as methyl attached to C-11a.

From the aforementioned data one can observe that compound **5** showed the same structure features of Saudin; a secolabdane previously isolated from *C. richardiana*; except in disappearance of the ether bridge between C-9a and C-5. On the basis of the above evidences, the structure of **5** could be named as 5-(fyran-3-yl)-3a’,7,11a-trimethyloctahydro-1H,3H-furo[3,4-f] pyrano [4,3,2-de] chromene-1,8-(3a’H)-dione and was given the trivial names Saudin-I.

### 2.2. Testing for Antibacterial Activity

It was found that none of the isolated compounds showed antibacterial activities against the tested pathogen.

### 2.3. Characterization of Possible ESBL Inhibitory Activities

**Figure 4:**
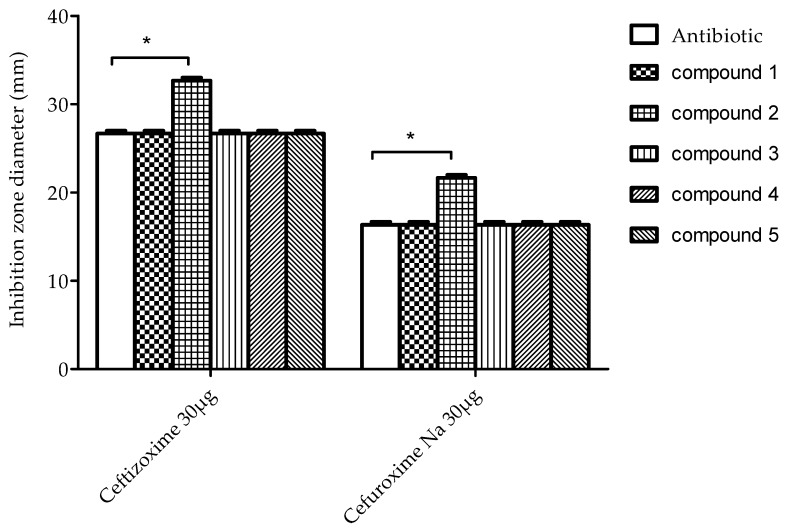
Antimicrobial susceptibility pattern against *K. pneumoniae* ATCC 700603 with and without addition of 10 μM of each compound. Data are presented as mean ± SE. * Significant interaction versus control (*p* < 0.01) determined by Student’s tests.

Compound **2** showed an obvious activity against *K. pneumoniae* ATCC 700603 with a marked enlargement of inhibition zones (>5 mm) in combination with second and third generation cephalosporin antibiotics (Cefuroxime Na 30 µg and Ceftizoxime 30 µg respectively) (Table 2 and Figure 4).

### 2.4. Molecular Modeling Study

**Figure 5:**
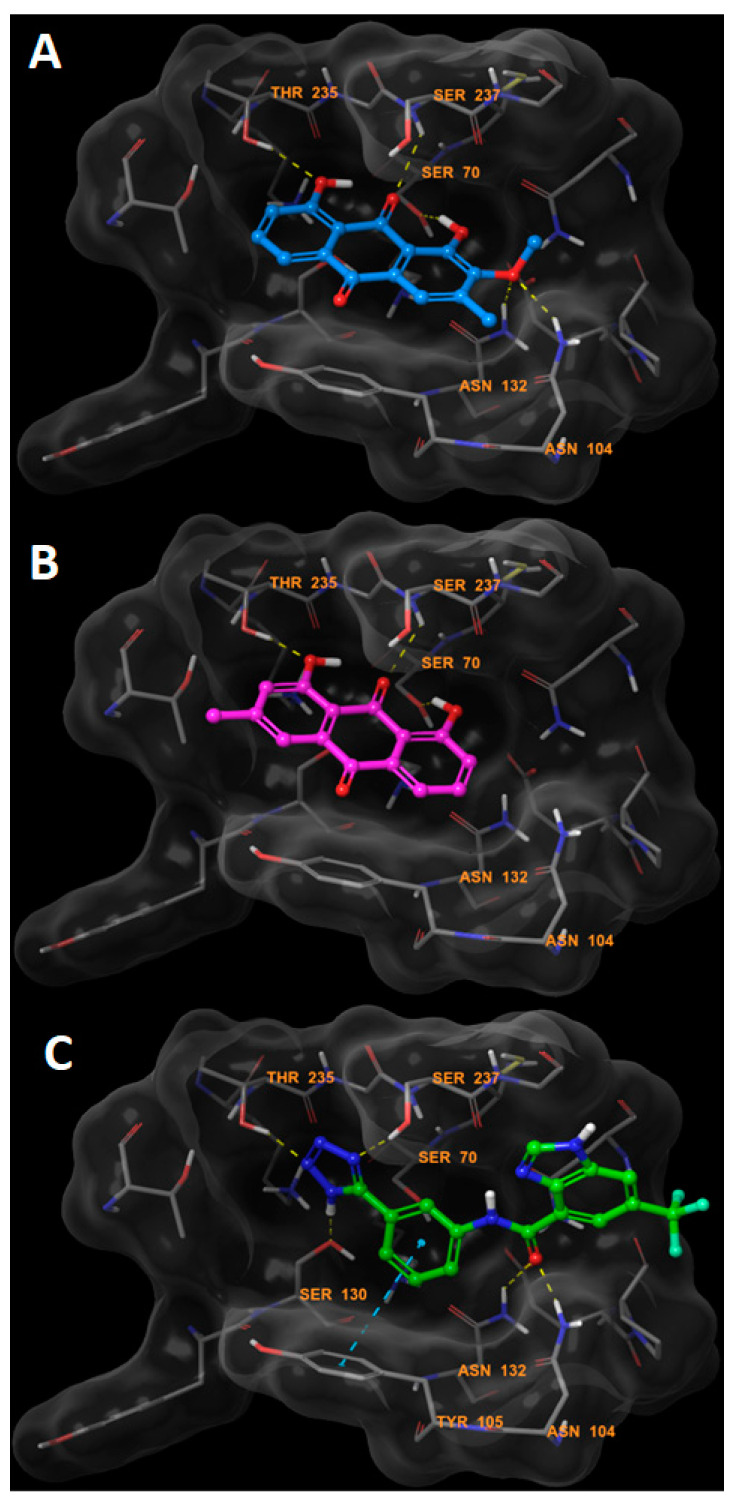
Binding modes of compound **2** (**A**) (cyan), compound **1** (**B**) (magenta) and the co-crystallized tetrazole inhibitor (**C**) (green).

**Figure 6:**
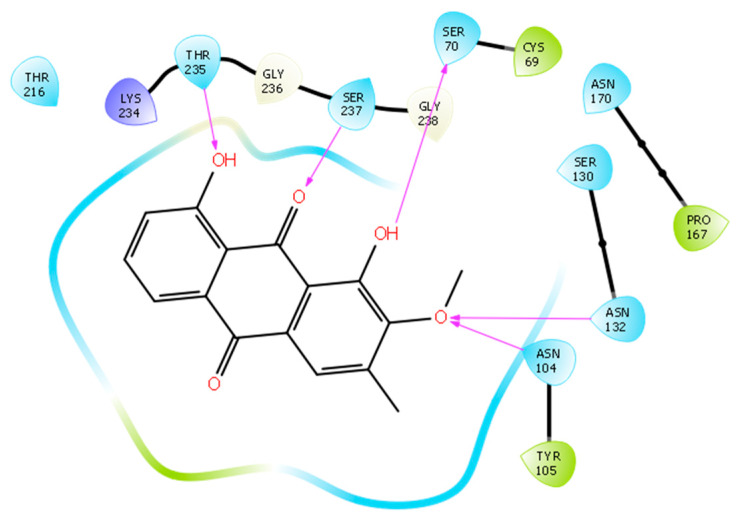
The 2D representation of the binding interaction of compound **2** in the binding pocket of CTX-M-27 (PDB ID: 6bu3).

To investigate the mechanism of action and binding of compound **2** to ESBL, we conducted a molecular docking study using Glide docking engine within the Schrodinger molecular modeling suite. The crystal structure of the CTX-M-27 beta lactamase co-crystallized with a non-covalent tetrazole inhibitor (PDB ID: 6bu3) was used as a receptor for ligand docking [28]. This co-crystal structure was particularly chosen since our inhibitor also potentially inhibits the beta-lactamase via a non-covalent mechanism because of the lack of reactive centers in its structure. A co-crystal structure of the enzyme with a non-covalent inhibitor was therefore chosen. Docking of this compound showed that it was able to form key interaction that are similar to those formed by the co-crystallized ligand. Compound **2** formed two hydrogen bonds with the side chains of Thr235 and Ser70 via its two phenolic hydroxyl groups. Despite its inability to form a hydrogen bond with the catalytic residue Ser237, the carbonyl group of compound **2** formed a compensatory hydrogen bond with the nitrogen atom of the backbone amide of this residue. An important interaction of compound **2** is that it is able to form two hydrogen bonds via its methoxy group with the side chains of Asn104 and Asn132. These hydrogen bonds are of special importance as they might explain the absence of activity of compound **1** despite having a structure very similar to compound **2**. Compound **1** lacks the methoxy group present in compound **2**, and hence there was no hydrogen bonding observed with Asn104 and Asn132 when it was docked into the binding pocket of CTX-M-27. Furthermore, compounds **3**–**5** lacked hydrogen bonding interactions that were observed in the poses of compound **2** and the co-crystallized tetrazole inhibitor. Binding mode and interactions of compounds **1** and **2** are represented in Figure 5 and Figure 6.

The mis-use of antibiotics resulted in the development of multidrug resistance against common anti-biotics that was considered as a challenge in the treatment of pathogenic micro-organisms. Among these pathogens are bacteria producing ESBL such as *P. aeruginosa, E. coli, K. pneumoniae,* and *A. baumannii.* Although, many well-known β-lactamase inhibitors are marketed yet, there is a critical need to a new and safer antimicrobial agent without cross-resistance as that available ones. Natural products are considered as a valuable source of antibacterial compounds. Previously, plant extracts of *Punica granatum* and *Delonix regia, Garcinia kola, Petalostigma* spp. and *Peganum harmala* showed high activity against β-lactamase [10,29,30]. Moreover, some isolated compounds showed promising β-lactamase inhibiting activity. Isoquinoline alkaloids from *Chelidonium majus* showed potent activity against ESBL-producing strains [31]. Also, phenolic compounds like that isolated from green tea (catechin gallate, epicatechin gallate, and epigallocatechin gallate), myricetin and anacardic acids from nuts of *Anacardium occidentale* demonstrated a potent β-lactamase inhibition [29,32,33]. 1,4-naphthoquinone, previously isolated from *Holoptelea integrifolia,* is very similar to active compound **2** and can inhibit the enzymatic activity of beta-lactamase in S. *aureus* [34]. Docking studies showed that 1,4-naphthoquinone is binding to the active site through only hydrogen bonds of its carbonyl group with Ser 70 and Lys 73 residues. It was observed that the main stabilizing factor of the complex is van der Waal’s interactions [34]. In this study, the method of binding of compound **2** (that follows anthraquinones) was more clearly presented. Its carbonyl group is also attached by compensatory H-bonding to the amide backbone, in addition; another two hydrogen bonds were observed between phenolic groups and Thr235 and Ser70. Moreover, methoxy group is important for activity because of its ability to bind with two hydrogen bonds with the side chains of Asn104 and Asn132.

## 3. Materials and Methods

### 3.1. General

HRESIMS was recorded on LTQ Orbitrap mass spectrometer (ThermoFinnigan, Bremen, Germany) equipped with a heated electrospray ion source (positive spray voltage 4 kV), capillary temperature of 300 °C, source heater temperature of 250 °C, scan range from 50 to 1600 *m/z*. 1D (^1^H-and ^13^C) and 2D NMR (HSQC, HMBC, NOESY, and COSY) spectra were recorded on Bruker DRX-850 and 600 MHz Ultrashield spectrometers (Bruker BioSpin, Billerica, MA, USA) using CDCl_3_ as solvent, with TMS as the internal reference. TLC analysis was performed on pre-coated TLC plates with silica gel 60 F_254_ (Merck, Darmstadt, Germany) using systems *n*-hexane:EtOAc (9.5:0.5, 9:1 and 8:2 *v/v*). Column chromatographic separations were performed on silica gel 60 (70–230 mesh, Merck, Darmstadt, Germany).

### 3.2. Plant Material

Total aerial parts of *Clutia myricoides* Jaub. & Spach (Euphorbiaceae) were collected from al Taif governorate. Plant was kindly identified by Dr. Emad Al-Sharif, Associate Professor of Plant Ecology, Department of Biology, Faculty of Science & Arts, Khulais, King Abdulaziz University, Saudi Arabia. A voucher specimen (CM-083B) was kept in the herbarium of faculty of pharmacy, King Abdulaziz University.

### 3.3. Extraction and Isolation

One kilogram of the dried aerial parts of *C*. *myricoides* (CM) were extracted with methanol (2.5 L × 4) using ultra turrax homogenizer at room temperature until exhaustion, and the collected extracts were evaporated under vacuum to give 150 g of brown residue. Total methanol extract (CMT) was suspended in the least amount of water and extracted with chloroform (300 mL × 3) that was evaporated under vacuum to give 40 g of dark brown residue. Chloroform fraction (40 g) was chromatographed on silica gel column (500 g, 100 × 4 cm) using *n*-hexane with gradual increasing of polarity using EtOAc from 9.5:0.5 to 6:4 *v/v*, to obtain eleven sub-fractions CM-1 to CM-11. Sub-fraction CM-2 (450 mg) was subjected to silica gel CC (20 g, 50 × 2 cm), eluted with *n*-hexane:EtOAc (99:1) to afford pure compound **1** (30 mg, red amorphous powder). Sub-fraction CM-6 (750 mg) was chromatographed on SiO_2_ CC (30 g, 50 × 2 cm), using *n*-hexane:EtOAc (95:5 till 8:2 *v/v*) to give two major compounds that were further purified on silica gel CC (10 g, 50 × 1 cm) to yield compound **2** (20 mg, yellow amorphous powder) and compound **3** (8 mg, white amorphous powder). Sub-fraction CM-7 (200 mg) contained one major spot that was purified on SiO_2_ CC (25 g, 50 × 2 cm), using *n*-hexane:EtOAc (80:20) to give compound **4** (10 mg, white amorphous powder). Fraction 11 (800 mg) was chromatographed over SiO_2_ CC (30 g, 50 × 2) using Hexane: EtOAc (8:2 *v/v*) to obtain compound **5** (7mg, white amorphous powder).

### 3.4. Spectral Data

2-methoxy chrysophanol (**2**): yellow amorphous powder; NMR data: see Table 1; HRESIMS *m*/*z* 285.0769 [M + H]^+^ (calcd for 285.0763, C_16_H_13_O_5_).

Saudin-I (**5**): White amorphous powder; [α]${}_{D}^{25}$ +71° (c 0.12, CHCl_3_); NMR data: see Table 1; HRESIMS *m*/*z* 361.1676 [M + H]^+^ (calcd for 361.1670, C_20_H_25_O_6_).

### 3.5. Bacterial Strain

*K. pneumoniae* ATCC 700603 was used in this study. Double-disc diffusion test was used to confirm phenotype and elucidations were recorded according to CLSI guidelines. Briefly, a disc containing 30 μg of ceftazidime and another containing a combination of 30 μg ceftazidime and 10 μg clavulanic acid (Mast, USA) were added, maintaining a distance of 30 mm, to the plates of Mueller-Hinton agar (Difco, USA) previously inoculated with a suspension of bacteria with a count adjusted by 0.5 McFarland tube (Difco, USA) then incubated at 37 °C for 24 hr. A ≥ 5-mm enlargement of inhibition zone diameter for the combination discs versus the ceftazidime discs confirmed extended spectrum β-lactamase production [35].

### 3.6. Screening for Antibacterial and Anti-ESBL Activity

Disc diffusion method was carried out for antibacterial testing [36]. Ten microliter (10 μM) of each isolated compound was added to a sterile disc with diameter of 6 mm (whatman^®^) and located on the inoculated agar. Negative control and positive control were performed using DMSO and suitable antibiotics correspondingly. The inoculated plates were incubated overnight at 37 °C. The antimicrobial activity was assessed by calculating the inhibition zone diameter. The assay was done thrice. For determination of the anti-ESBL of isolated compounds with antibiotic, the antibiotic and antibiotic loaded with 10 μL (10 μM) of each isolated compound discs were added at a distance of 30 mm on plates of Mueller-Hinton agar inoculated with *K. pneumoniae* ATCC 700603, then incubated at 37 °C for 24 hr. A ≥ 5-mm enlargement of inhibition zone indicates a positive interaction [37].

### 3.7. Molecular Modeling

**Figure 7:**
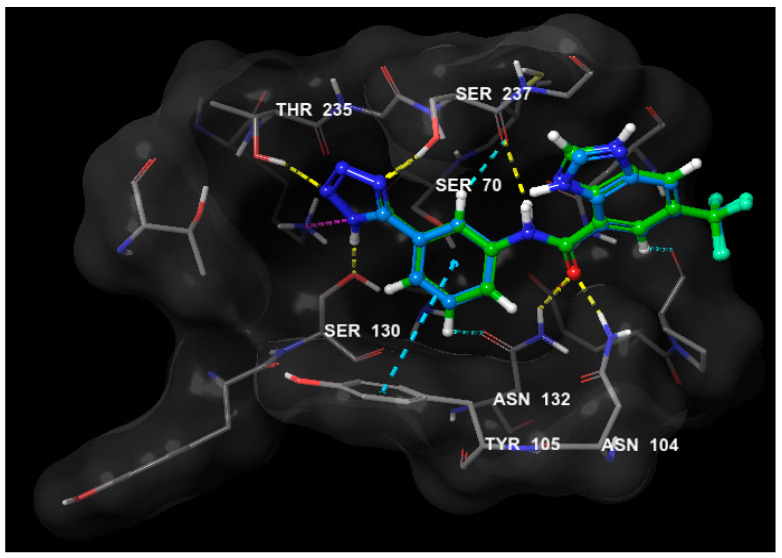
3D overlay of the co-crystallized tetrazole (green) inhibitor and its re-docked pose (cyan) in the binding site of CTX-M-27 showing minimum deviation of the docked pose from the co-crystallized one (RMSD = 0.0878 Å) and therefore validating the docking protocol.

In the docking experiment, Glide docking engine within the Schrödinger Suite was utilized. All ligands were prepared using Ligprep where ligands were typed with OPLS3 force field and ionization states within pH range 7 +/− 2 were generated. The CTX-M-27 crystal structure co-crystalized with a tetrazole inhibitor was obtained from the Protein Data Bank (PDB ID: 6bu3) and prepared with the protein preparation wizard of Schrödinger Suite where water molecules were deleted, protein was typed with OPLS3 force field and minimized. Next, a receptor grid was generated using the co-crystalized ligand as reference. The docking protocol used flexible ligand sampling and standard precision with no docking constraints. Docking scores for compounds **1**–**5** were −4.986, −5.544, −4.572, −4.843, and −4.916 respectively. To validate the docking protocol, the co-crystalized inhibitor was re-docked using the same docking parameters and the RMSD calculated to be 0.0878Å (Figure 7).

## 4. Conclusions

In this work, antibacterial and anti ESBL activity of six compounds were performed, where no antibacterial activities were detected for any of the tested compounds. Meanwhile, compound **2** showed promising anti ESBL activity. These results support the concomitant use of this compound with antibiotics to increase its efficiency. Nevertheless, the interaction between active compound and antibiotics should be taken into consideration. Nonetheless, in order to evaluate the safety of these compounds further in vitro and in vivo toxicity assays must be carried out.

## Figures and Tables

**Table 1:** NMR spectral data of isolated compounds **2** and **5** (CDCl_3_, 850 and 212.5 MHz).

No.	2			5	
	δ_H_ [Mult. *J* (Hz)]	δ_C_		δ_H_ [Mult. *J* (Hz)]	δ_C_
**1**	-	159.7	1	-	178.0
**2**	-	166.4	3	4.18 d (9.4), 4.28 d (9.4)	70.4
**3**	-	146.4	3a	-	82.0
**4**	7.69 s	121.5	3a′	-	35.2
**4a**	-	129.0	5	5.11 d (1.7)	92.0
**4b**	-	133.1	6	1.25 m, 1.28 m	29.7
**5**	7.83 dd (1.7,7.6)	120.3	6a	2.70 m	38.4
**6**	7.70 d (7.6)	137.4	7	3.02 m	35.3
**7**	7.31 dd (1.7,7.6)	124.9	8	-	173.0
**8**	-	162.6	9	-	82.0
**8a**	-	115.7	9a	4.37 dd (4.2,11.9)	75.5
**8b**	-	114.1	10	1.78 m, 1.84 m	23.9
**9**	-	192.4	11	1.62 m, 2.42 m	26.5
**10**	-	181.4	12	1.41 d (6.8)	13.7
OCH_3_	3.99 s	52.8	13	1.20 s	15.3
CH_3_	2.44 s	20.1	14	1.43 s	23.0
OH-1	12.37	-	2′	7.54 brs	139.4
OH-8	11.90	-	3′	-	121.0
			4′	6.45 brs	107.0
			5′	7.40 brs	144.8

**Table 2:** Antimicrobial susceptibility pattern against *K. pneumoniae* ATCC 700603 with and without addition of 10 μM of each compound.

Code	Antibacterial/Anti ESBL Activity
**1**	−/−
**2**	−/+ *
**3**	−/−
**4**	−/−
**5**	−/−

*a > 5-mm increase in the diameter of inhibition zone for the combination disc, 10 μM with Ceftizoxime 30 µg and Cefuroxime Na 30 µg.

## Supplementary Materials

Supplementary materials are available online.

## References

[1] Lee K., Chong Y., Shin H., Kim Y., Yong D., Yum J. (2001). Modified Hodge and EDTA-disk synergy tests to screen metallo-β-lactamase-producing strains of Pseudomonas and Acinetobactet species. Clin. Microbiol. Infect..

[2] Kaur M., Aggarwal A. (2013). Occurrence of the CTX-M, SHV and the TEM Genes among the extended spectrum β-Lactamase producing isolates of enterobacteriaceae in a tertiary care hospital of North India. J. Clin. Diagn. Res..

[3] Hujer A.M., Hujer K.M., Helfand M.S., Anderson V.E., Bonomo R.A. (2002). Amino acid substitutions at Ambler position Gly238 in the SHV-1 β-lactamase: Exploring sequence requirements for resistance to penicillins and cephalosporins. Antimicrob. Agents Chemother..

[4] Wright G.D. (2005). Bacterial resistance to antibiotics: Enzymatic degradation and modification. Adv. Drug Deliv. Rev..

[5] Chen J., Shang X., Hu F., Lao X., Gao X., Zheng H., Yao W. (2013). β-Lactamase inhibitors: An update. Mini Rev. Med. Chem..

[6] Fenical W., Jensen P.R. (2006). Developing a new resource for drug discovery: Marine actinomycete bacteria. Nat. Chem. Biol..

[7] Gangoué-Piéboji J., Baurin S., Frère J.M., Ngassam P., Ngameni B., Azebaze A., Pegnyemb D.E., Watchueng J., Goffin C., Galleni M. (2007). Screening of some medicinal plants from Cameroon for β-lactamase inhibitory activity. Phytother. Res..

[8] Nascimento G.G., Locatelli J., Freitas P.C., Silva G.L. (2000). Antibacterial activity of plant extracts and phytochemicals on antibiotic-resistant bacteria. Braz. J. Microbiol..

[9] Zhao W.-H., Hu Z.-Q., Okubo S., Hara Y., Shimamura T. (2001). Mechanism of synergy between epigallocatechin gallate and β-lactams against methicillin-resistant Staphylococcus aureus. Antimicrob. Agents Chemother..

[10] Aqil F., Khan M.S.A., Owais M., Ahmad I. (2005). Effect of certain bioactive plant extracts on clinical isolates of β-lactamase producing methicillin resistant *Staphylococcus aureus*. J. Basic Microbiol..

[11] Asili J., Emami S.A., Eynolghozat R., Noghab Z.S., Bazzaz B.S.F., Sahebkar A. (2015). Chemical Composition and *In Vitro* Efficacy of Essential Oil of Seven *Artemisia* Species Against ESBL Producing Multidrug-Resistant *Escherichia coli*. J. Essent. Oil Bear. Plants.

[12] Lin R.D., Chin Y.P., Lee M.H. (2005). Antimicrobial activity of antibiotics in combination with natural flavonoids against clinical extended-spectrum β-lactamase (ESBL)-producing Klebsiella pneumoniae. Phytother. Res..

[13] Parveen M., Ahmad F., Malla A.M., Azaz S., Alam M., Basudan O.A., Silva M.R., Silva P.S.P. (2016). Acetylcholinesterase and cytotoxic activity of chemical constituents of *Clutia lanceolata* leaves and its molecular docking study. Nat. Prod. Bioprospect..

[14] Mossa J.S., Cassady J.M., Kozlowski J.F., Zennie T.M., Antoun M.D., Pellechia M.G., McKenzie A.T., Byrn S.R. (1988). Novel 6, 7-seco-6, 11-cyclolabdane diterpenes from *Cluytia richardiana*. Tetrahedron Lett..

[15] Muhammad I., Mossa J.S., Al-Yahya M.A., Mirza H.H., El-Feraly F.S., Mcphail A.T. (1994). Modified labdane diterpenoids from *Cluytia richardiana*. Phytochemistry.

[16] Muhammad I., Mossa J.S., Mirza H.H., El-Feraly F.S. (1999). A new modified 6, 7-secolabdane diterpenoid from *Clutia richardiana*. Phytochemistry.

[17] Muhammad I., Mossa J.S., Al-Yahya M.A., Mirza H.H., El-Feraly F.S., McPhail A.T. (1994). New modified labdane diterpenoids from *Cluytia richardiana*. J. Nat. Prod..

[18] Mossa J.S., Cassady J.M., Antoun M.D., Byrn S.R., McKenzie A.T., Kozlowski J.F., Main P. (1985). Saudin, a hypoglycemic diterpenoid, with a novel 6, 7-seco-labdane carbon skeleton, from *Cluytia richardiana*. J. Org. Chem..

[19] Mossa J.S., Muhammad I., Al-Yahya M.A., Mirza H.H., El-Feraly F.S., McPhail A.T. (1996). Five new modified 6, 7-secolabdane diterpenoids from *Cluytia richardiana*. J. Nat. Prod..

[20] Waigh R.D., Zerihun B., Melvin R. (1990). Three diterpenes with a secolabdane skeleton from *Clutia abyssinica*. Phytochemistry.

[21] Waigh R.D., Zerihun B.M., Maitland D.J. (1991). Ten 5-methylcoumarins from *Clutia abyssinica*. Phytochemistry.

[22] Abdallah H.M., Asfour H.Z., El-Halawany A.M., Elfaky M.A. (2018). Saudi plants as a source of potential β-lactamase inhibitors. Pak. J. Pharm. Sci..

[23] Albar H.A., Alsofuani T.A., Khorshed F.A. Phytochemical Analysis and Biological Screening of Leaf Extracts from *Clutia myricoides*. Proceedings of the Oryx Conference for the Life Sciences.

[24] El-Halawany A.M., Chung M.H., Nakamura N., Ma C.-M., Nishihara T., Hattori M. (2007). Estrogenic and anti-estrogenic activities of *Cassia tora* phenolic constituents. Chem. Pharm. Bull..

[25] Zamzami T.A., Abdallah H.M., Shehata I.A., Mohamed G.A., Alfaifi M.Y., Elbehairi S.E.I., Koshak A.E., Ibrahim S.R. (2019). Macrochaetosides A and B, new rare sesquiterpene glycosides from *Echinops macrochaetus* and their cytotoxic activity. Phytochem. Lett..

[26] El-Halawany A.M., Osman S.M., Abdallah H.M. (2019). Cytotoxic constituents from *Vicia monantha* subsp. monantha seeds. Nat. Prod. Res..

[27] Nair M.G.D., Mugunthu R., Kron M.A., Milev Y.P. (2010). Anthraquinones and Process for the Preparation and Method of Use Thereof. US Patents.

[28] Pemberton O.A., Zhang X., Nichols D.A., DeFrees K., Jaishankar P., Bonnet R., Adams J., Shaw L.N., Renslo A.R., Chen Y. (2018). Antibacterial spectrum of a tetrazole-based reversible inhibitor of serine β-lactamases. Antimicrob. Agents Chemother..

[29] Cheesman M.J., Ilanko A., Blonk B., Cock I.E. (2017). Developing new antimicrobial therapies: Are synergistic combinations of plant extracts/compounds with conventional antibiotics the solution?. Pharmacogn. Rev..

[30] Saeidi S., Boroujeni N.A., Ahmadi H., Hassanshahian M. (2015). Antibacterial activity of some plant extracts against extended-spectrum beta-lactamase producing Escherichia coli isolates. Jundishapur J. Microbiol..

[31] Zuo G.-Y., Meng F.-Y., Han J., Hao X.-Y., Wang G.-C., Zhang Y.-L., Zhang Q. (2011). In vitro activity of plant extracts and alkaloids against clinical isolates of extended-spectrum β-lactamase (ESBL)-producing strains. Molecules.

[32] Abreu A.C., McBain A.J., Simoes M. (2012). Plants as sources of new antimicrobials and resistance-modifying agents. Nat. Prod. Rep..

[33] Bouttier S., Fourniat J., Garofalo C., Gleye C., Laurens A., Hocquemiller R. (2002). β-Lactamase Inhibitors from Anacardium occidentale. Pharm. Biol..

[34] Vinod N., Shijina R., Dileep K., Sadasivan C. (2010). Inhibition of beta-lactamase by 1, 4-naphthalenedione from the plant *Holoptelea integrifolia*. Appl. Biochem. Biotechnol..

[35] Coudron P.E., Moland E.S., Sanders C.C. (1997). Occurrence and detection of extended-spectrum beta-lactamases in members of the family Enterobacteriaceae at a veterans medical center: Seek and you may find. J. Clin. Microbiol..

[36] Murray P., Baron E., Pfaller M., Tenover F., Yolke R. (1995). Manual of Clinical Microbiology.

[37] Chattopadhyay D., Dastidar S., Chakrabarty A. (1988). Antimicrobial properties of methdilazine and its synergism with antibiotics and some chemotherapeutic agents. Arzneimittelforschung.

