# Supplementary material for: Bioassay Guided Isolation and Docking Studies of a Potential β-Lactamase Inhibitor from Clutia myricoides
Source: Molecules. 2020 May 31;25(11):2566. doi: 10.3390/molecules25112566 (PMC7321312; doi:10.3390/molecules25112566)
Supplement: Supplementary file 1 [file molecules-25-02566-s001.pdf]

Bioassay Guided Isolation and Docking studies of a Potential β-Lactamase Inhibitor from *Clutia myricoides*

Mahmoud A. Elfaky[1], Ali M. El-Halawany[2], Abdulrahman E Koshak[1]

Khalid Z Alshali[3], Moustafa El-Araby[4] and Hossam M. Abdallah[1,2,*]

[1] Department of Natural Products, Faculty of Pharmacy, King Abdulaziz University, Jeddah 21589, Kingdom of Saudi Arabia
[2] Department of Pharmacognosy, Faculty of Pharmacy, Cairo University, Cairo 11562, Egypt
[3] Department of Medicine, Faculty of Medicine, King Abdulaziz University, Jeddah, Saudi Arabia
[4] Department of Medicinal Chemistry, Faculty of Pharmacy, King Abdulaziz University, Jeddah 21589, Kingdom of Saudi Arabia

* Correspondence: Dr. Hossam M. Abdallah; email: hmafifi2013@gmail.com, Tel.: +966-544-733-110

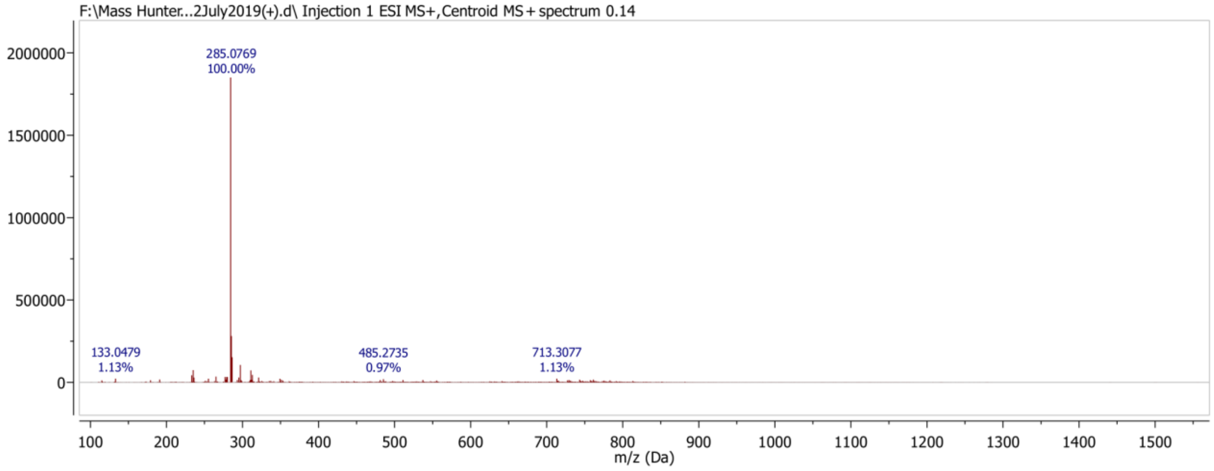

Figure S1: HRESIMS spectrum of compound **2**.

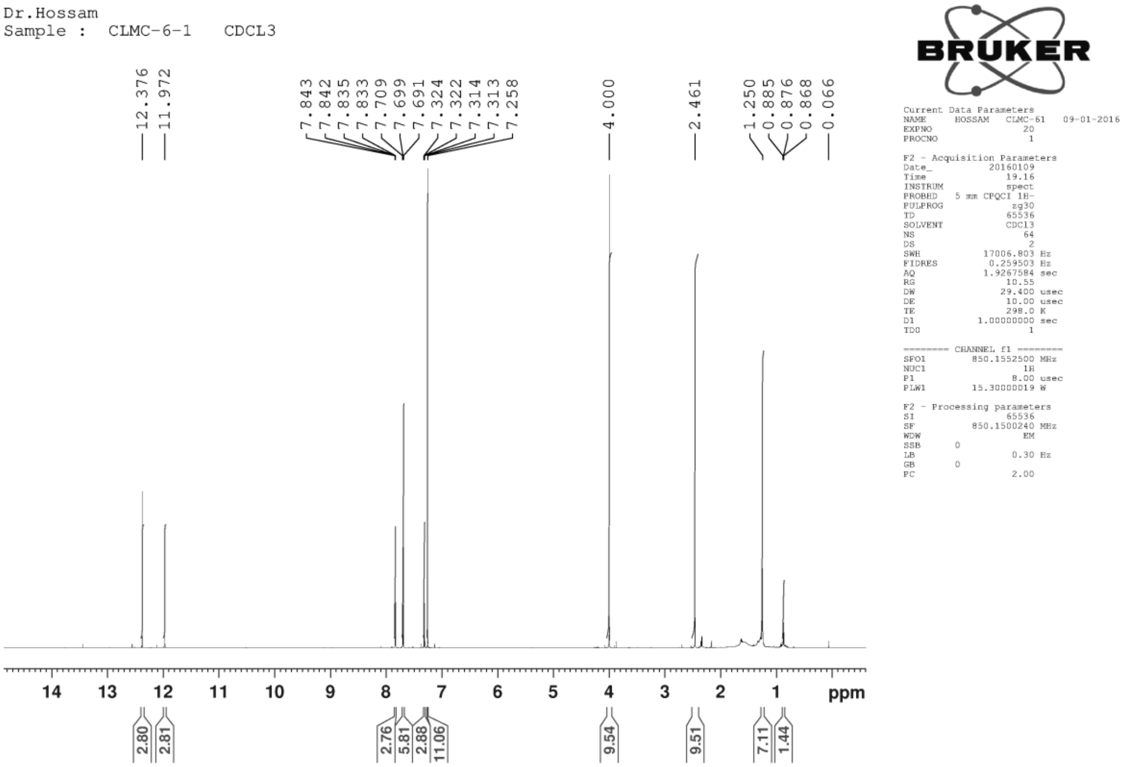

Figure S2: ^{1}H NMR spectrum of compound **2** (850 MHz, $CDCl_3$).

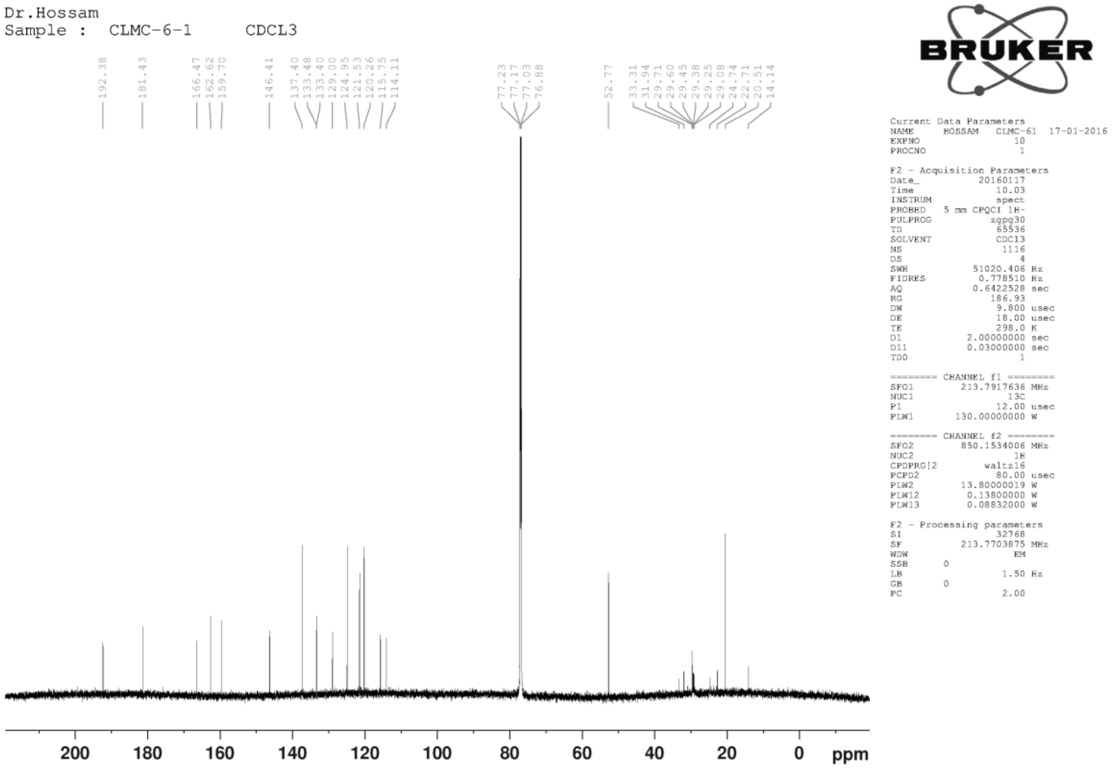

Figure S3: ^{13}C NMR spectrum of compound **2** (214 MHz, $CDCl_3$).

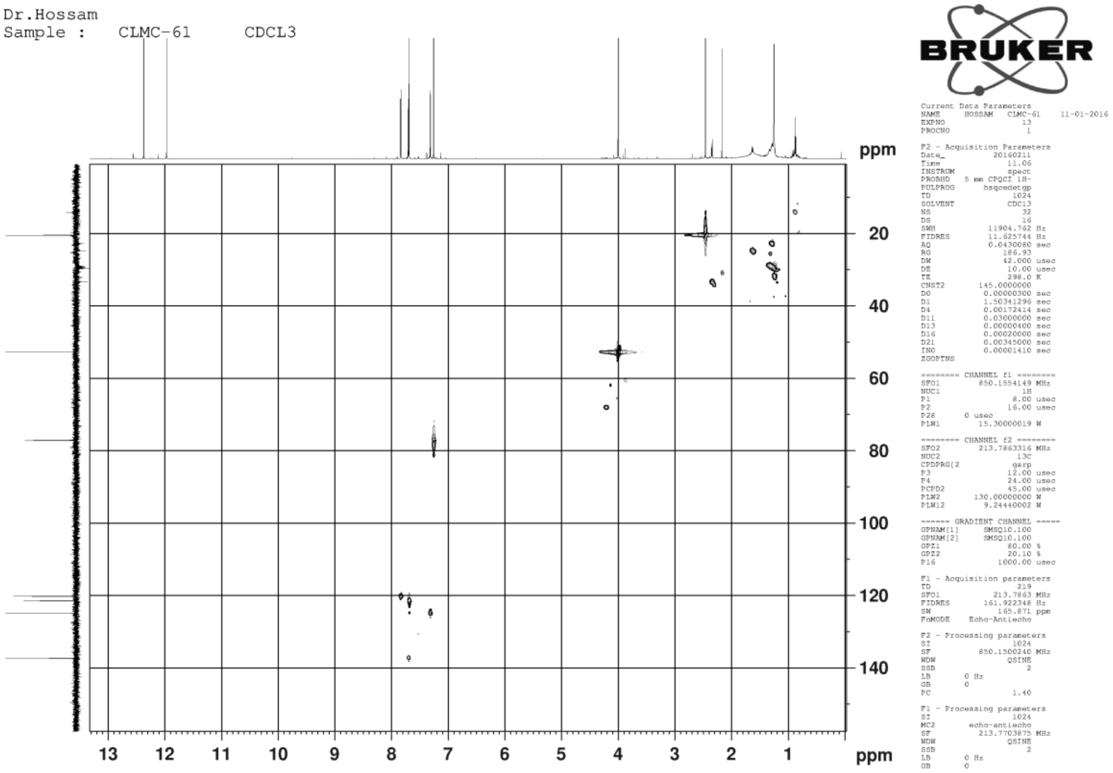

Figure S4: HSQC spectrum of compound **1** ($CDCl_3$).

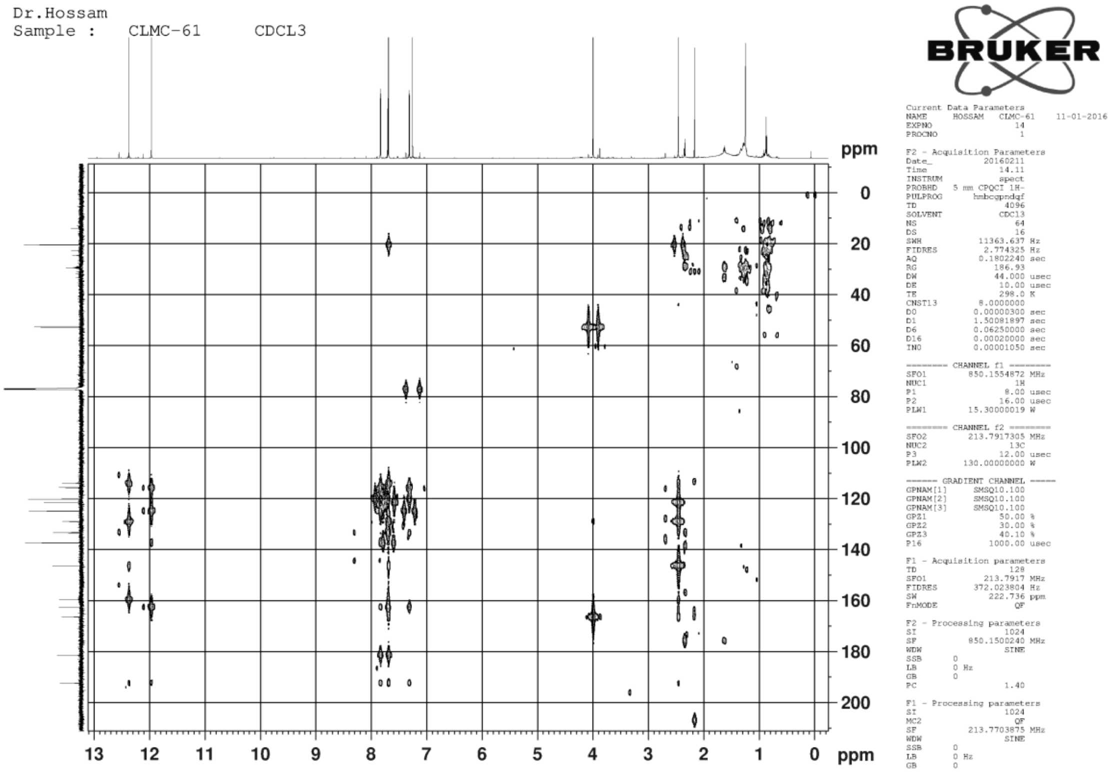

Figure S5: HMBC spectrum of compound **1** ($CDCl_3$).

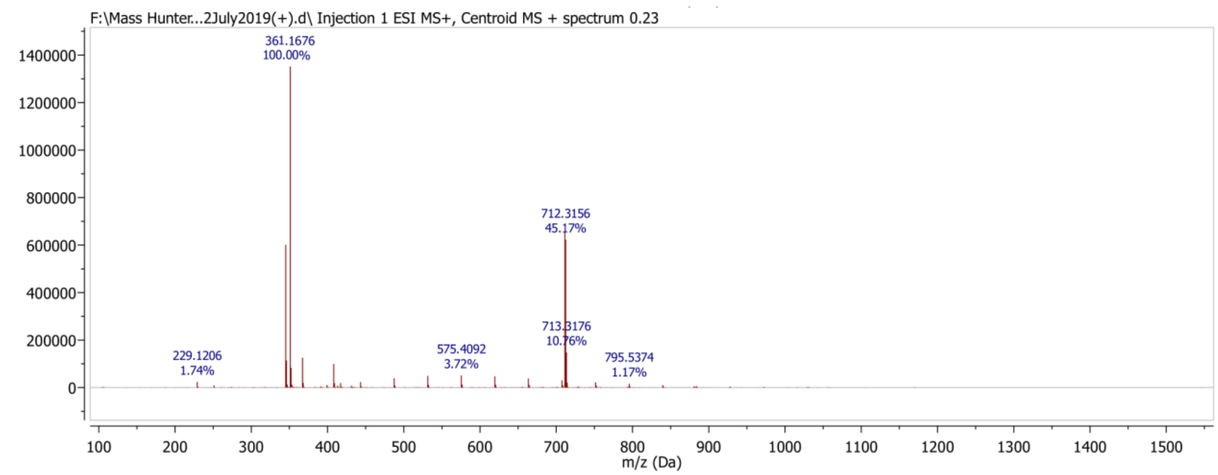

Figure S6: HRESIMS spectrum of compound **5**.

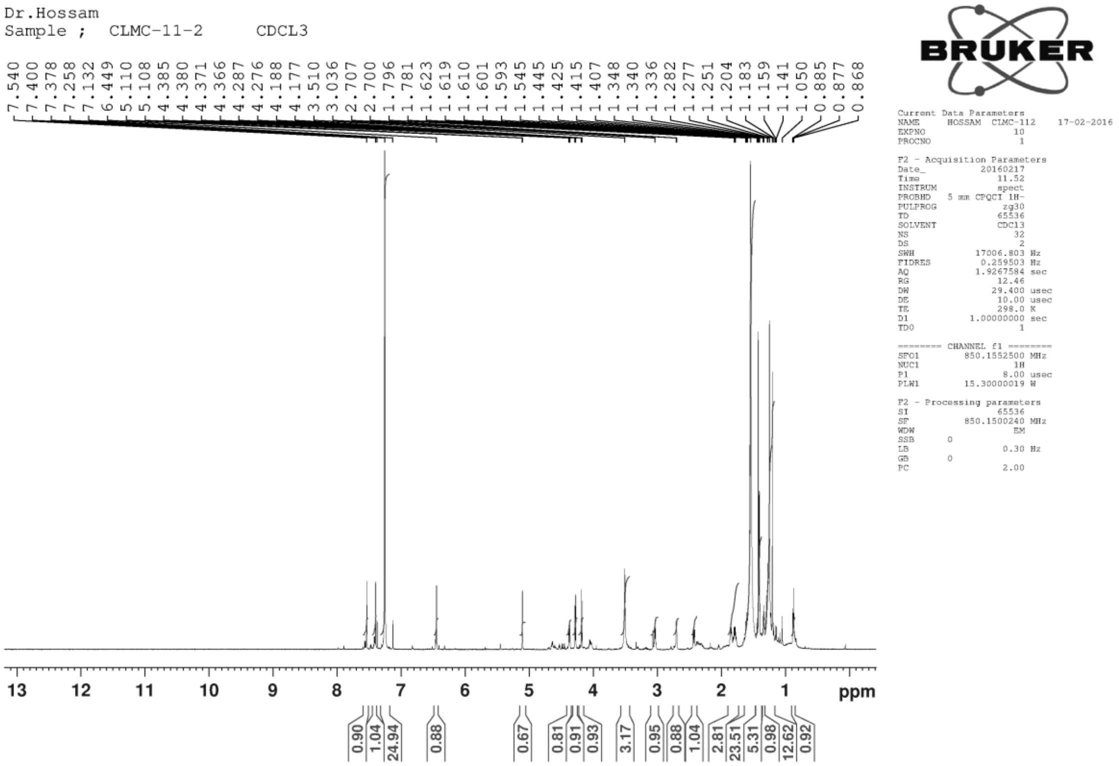

Figure S7. **^{1}H NMR spectrum of compound 5 (850 MHz, $CDCl_3$).**

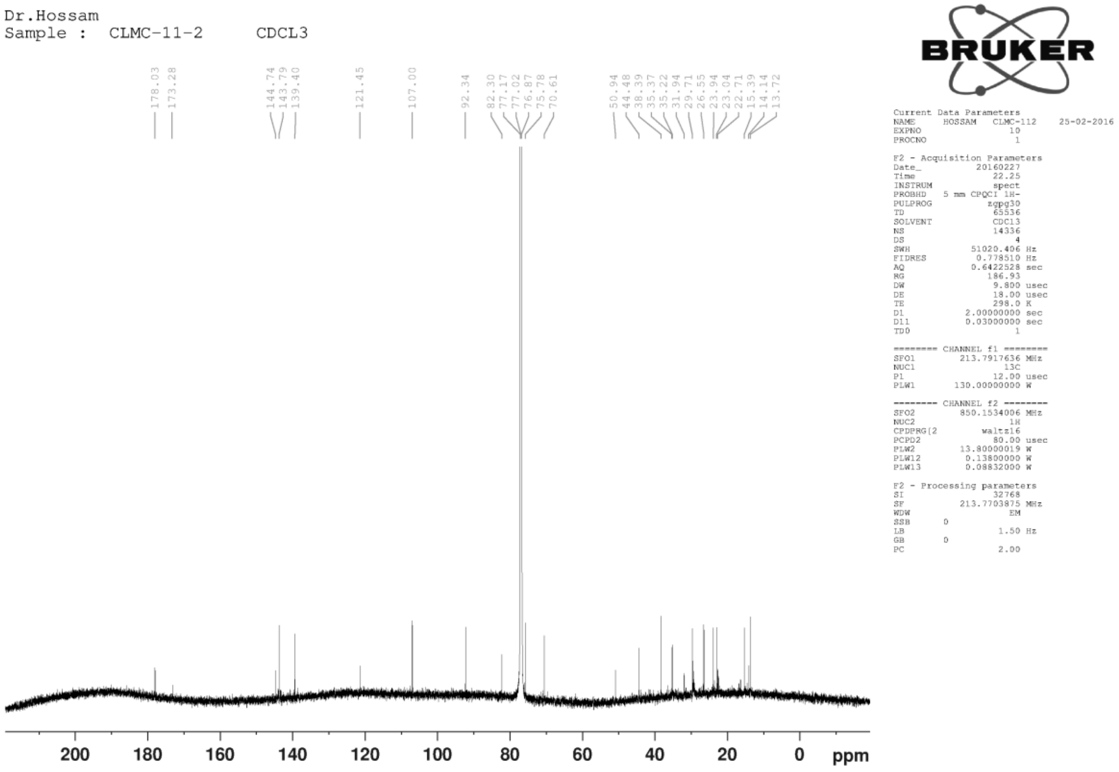

Figure S8: ^{13}C NMR spectrum of compound 5 (214 MHz, $CDCl_3$).

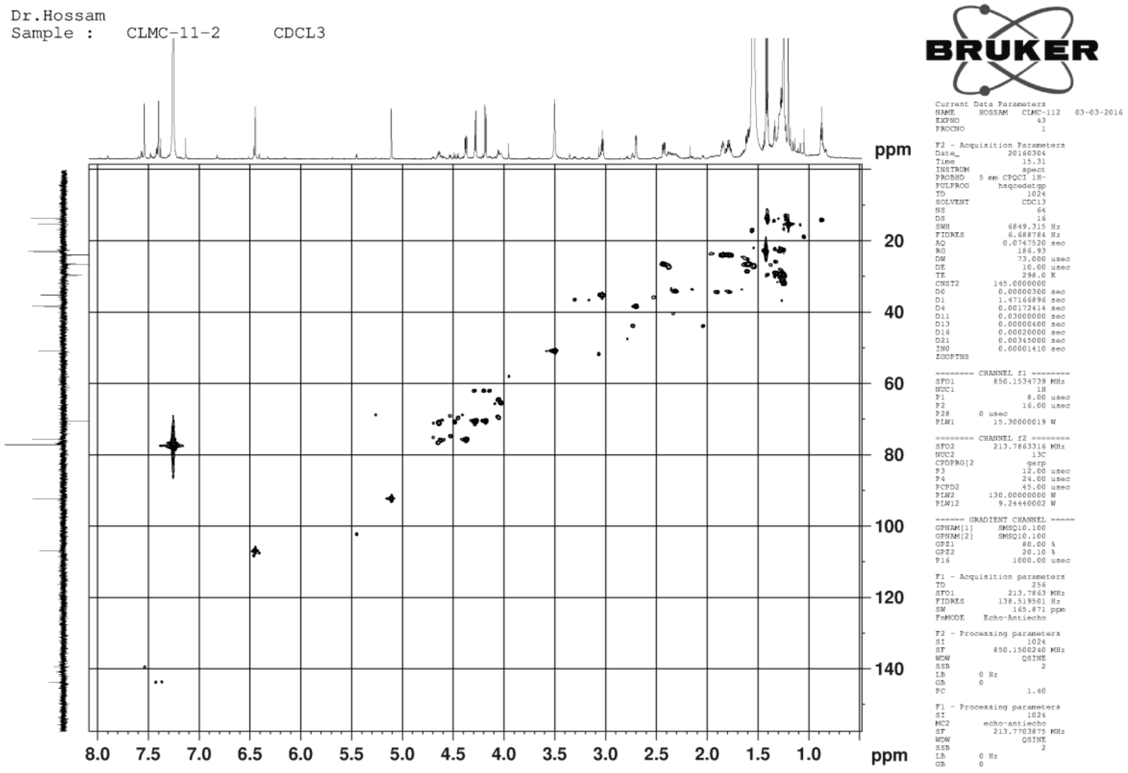

Figure S9: HSQC spectrum of compound 5 ($CDCl_3$).

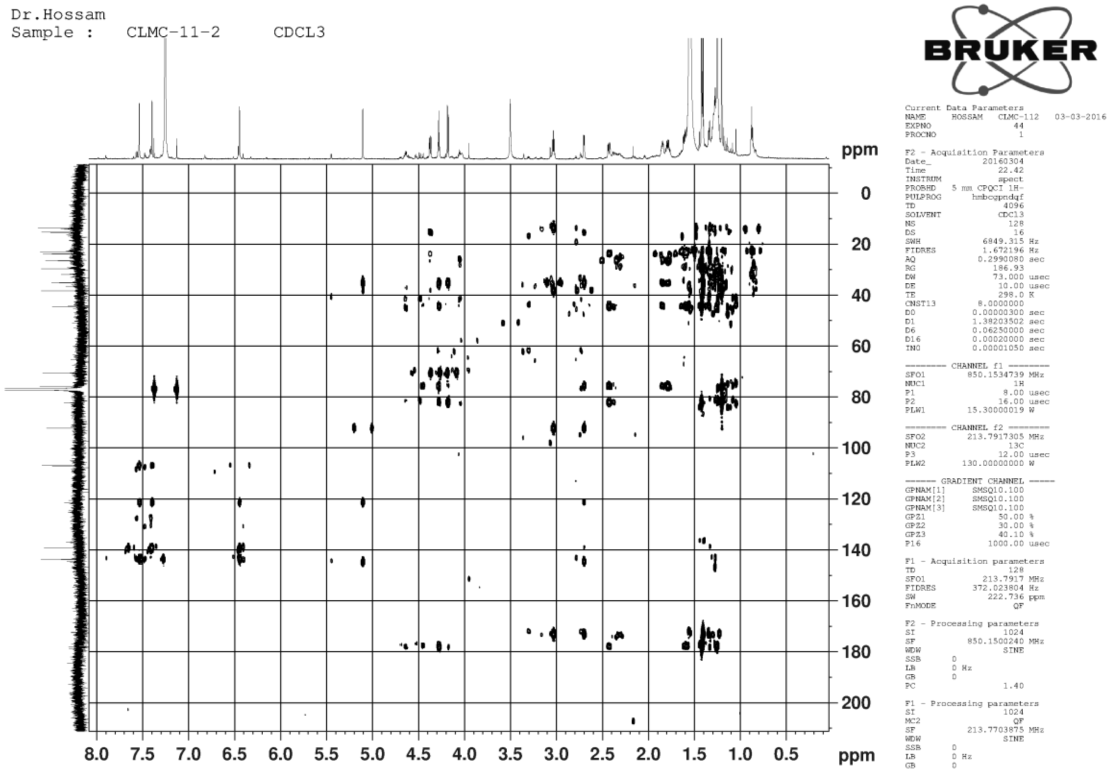

Figure S10: HMBC spectrum of compound 5 ($CDCl_3$).

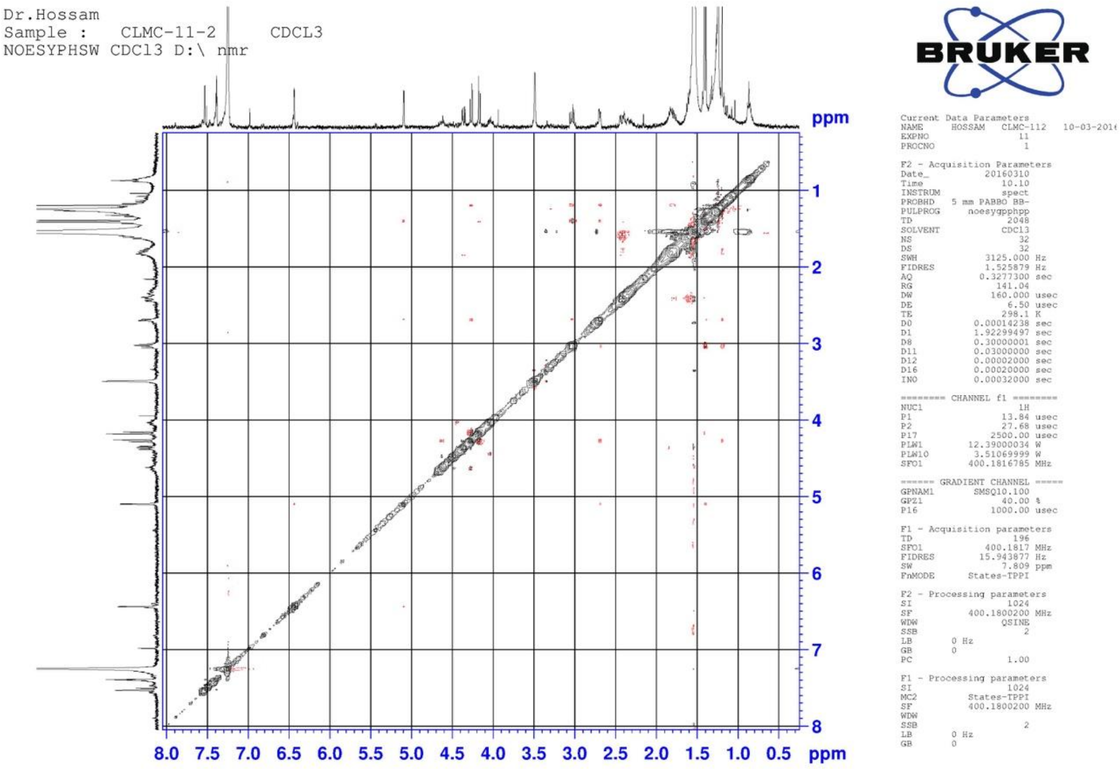

Figure S11: NOESY spectrum of compound 5 ($CDCl_3$).
